# Dysphagia Characteristics in High Versus Low Vagal Unilateral Vocal Fold Paralysis

**DOI:** 10.1002/lary.70229

**Published:** 2025-10-30

**Authors:** Madeline Miles Marker, Liam W. Gallagher, Aanish Puri, Jesse Hoffmeister, Scott Lunos, Heather Erickson, Gina Cruciani, Joslyn Kahn, Stephanie Misono, Raluca Gray

**Affiliations:** 1 Department of Otolaryngology-Head and Neck Surgery, University of Minnesota, Minneapolis, Minnesota, USA; 2 Biostatistical Design and Analysis Center, Clinical and Translational Science Institute, University of Minnesota, Minneapolis, Minnesota, USA; 3 M Health Fairview Rehab Services, Minneapolis, Minnesota, USA

**Keywords:** dysphagia, level of vagal injury, unilateral vocal fold paralysis

## Abstract

**Objective:**

To compare instrumental swallow assessment findings and diet recommendations in high versus low vagal unilateral vocal fold paralysis (UVFP).

**Methods:**

Retrospective review of patients with UVFP who underwent instrumental swallow assessment, September 2019–February 2024. Demographics, Eating Assessment Tool-10 (EAT-10) score, flexible laryngoscopy findings, instrumental swallow parameters, diet recommendations, treatment modalities, and posttreatment outcomes were analyzed.

**Results:**

Ninety-six patients were included: 28 (29%) high-vagal and 68 (71%) low-vagal UVFP. High vagal UVFP had a higher incidence of premature spillage (57% vs. 13%, *p* < 0.0001); residue (82% vs. 22%, *p* < 0.0001), penetration (89% vs. 35%, *p* < 0.0001), aspiration (50% vs. 22%, *p* = 0.013), modified diet (61% vs. 16%, *p* < 0.0001), and behavioral modifications (89% vs. 38%, *p* < 0.001) compared to low vagal UVFP. Thirty-one patients (32%) underwent injection laryngoplasty (16 high, 15 low vagal) with similar pretreatment prevalences of premature spillage, penetration, and aspiration, but a higher prevalence of residue in the high vagal group (100% vs. 53%, *p* = 0.002). Both groups improved posttreatment (high vagal: 63%–19%, *p* = 0.016; low vagal: 80%–7%, *p* = 0.001).

**Conclusion:**

High vagal UVFP is associated with greater swallowing dysfunction and higher prevalences of diet and behavioral modifications compared to low vagal UVFP. Treated high and low vagal subgroups had similar dysphagia profiles. Injection laryngoplasty improved aspiration, regardless of vagal level, although many patients continued to require behavioral modifications. Future studies are needed to identify predictors of poor functional swallowing outcomes in UVFP.

**Level of Evidence:**

3.

## 1 | Introduction

Dysphagia prevalence in unilateral vocal fold paralysis (UVFP) ranges widely (3%–69%) [1, 2], influenced by variability in swallow impairment based on the level of vagal injury and inconsistent definitions of dysphagia. Low vagal injury (isolated recurrent laryngeal nerve [RLN] damage) results in (1) glottal insufficiency with the inability to effectively close the glottis and protect the airway during swallowing and (2) impairment of the inferior pharyngeal constrictor muscles and upper cervical esophagus [3]. High vagal injury may also involve the superior laryngeal nerve (SLN) and branches to the pharynx, and may lead to broader deficits due to (1) decreased sensation to the larynx and pharynx, (2) weakened pharyngeal contractility, (3) velopharyngeal insufficiency, and (4) discoordination between the oral and pharyngeal phases with delayed swallow initiation [4]. Definitions of dysphagia vary to include (1) patient-reported concerns; (2) patient-reported outcome measures (e.g., elevated Eating Assessment Tool-10 [EAT-10] score); (3) abnormalities on instrumental swallow assessments (e.g., flexible endoscopic evaluation of swallowing [FEES] or modified barium swallow study/videofluoroscopic swallow study [MBSS/VFSS]); and (4) need for diet and meal-time behavioral modifications.

Prior studies on high versus low vagal UVFP were limited by small cohort size [5, 6], evaluation of a single dysphagia characteristic such as aspiration [4, 7–9] and/or incomplete characterization of the impact of vagal injury level on clinical and functional patient outcomes [10, 11]. Variable dysphagia definitions also make evaluation of treatment outcomes for low versus high vagal UVFP complex. Some studies show inconclusive improvement in aspiration after injection laryngoplasty [12, 13], while others show definitive improvement in oral intake [14]. The level of vagal injury is a potential confounding factor in prior studies. Some small studies reported worse outcomes in patients with high vagal paralysis [10, 15, 16], while others had limited information on vagal level within the analysis [17]. To date, no study has directly compared treatment responses between high and low vagal UVFP.

The aim of this study was to provide a comprehensive comparison of swallowing dysfunction in patients with high versus low vagal UVFP, as defined by (1) instrumental swallow assessment parameters and (2) patient-centered outcomes of diet and need for behavioral modifications. We hypothesized that the high vagal UVFP group would have worse swallow dysfunction. On an exploratory basis, we aimed to evaluate the impact of injection laryngoplasty on these outcomes.

## 2 | Methods

### 2.1 | Participants

This retrospective review was approved by the Institutional Review Board at the University of Minnesota (STUDY00021539). Medical records from the laryngology clinic from September 1, 2019 to February 29, 2024 were reviewed for patients over 18 years of age with UVFP who underwent instrumental swallow testing. UVFP was identified by ICD10 codes (ICD-9–478.30: paralysis of vocal cords or larynx, unspecified; ICD-10-J38.01: paralysis of vocal cords and larynx: unilateral, partial; ICD-10-J38.0: paralysis of vocal cords and larynx). Inclusion criteria required completion of instrumental swallow testing via FEES or MBSS/VFSS after the onset of vocal fold paralysis. Patients were excluded if they had bilateral vocal fold paralysis, a history of head and neck radiation therapy, a history of neurodegenerative diagnosis and/or the presence of a tracheostomy tube.

### 2.2 | Data Collection

Using a secure REDCap platform [18], pertinent data were collected including demographic data (age, sex, race), patient comorbidities (Charlson Comorbidity Index) [19], EAT-10 score, physical exam findings (presence of oral cavity neurologic deficits), flexible laryngoscopy findings (side of paralysis, presence of glottal gap, pyriform sinus pooling of saliva). To minimize potential confounding by nerve function recovery on the dysphagia findings, the flexible laryngoscopy findings at the time of office evaluation closest to swallow assessment were included in the analysis, which allowed confirmation of persistent paralysis. Both the duration of paralysis, as calculated from the otolaryngology clinic encounter, and the timing of the objective swallow testing relative to the onset of vocal fold paralysis, were noted for analysis. Similarly, in the subgroup of patients who received treatment, care was taken to exclude those who had recovered vocal fold movement at the time of posttreatment swallow testing.

Vocal fold paralysis characteristics were noted, including the duration of vocal fold paralysis, and the etiology of vocal fold paralysis (cerebrovascular accident [CVA], tumor compression, idiopathic, intubation, or iatrogenic [due to neurosurgery with craniotomy, esophageal surgery, neck surgery, or thoracic surgery]). Based on the etiology of paralysis identified from the otolaryngology notes, the patients were divided into low and high vagal injury based on anatomic cut-off (with low vagal injury due to isolated RLN involvement and high vagal injury due to injury above the level of the bifurcation of the RLN). The following categories of etiologies were included in the high vagal group:

- Neurological etiology (CVA).
- Tumor (skull base tumor, metastatic mass causing compression at the skull base and jugular foramen, cerebellopontine angle tumors).
- Iatrogenic (surgery involving the skull base/craniotomy, surgery along the main trunk of the vagus nerve to include paraganglioma and carotid body tumor surgery, vagal schwannoma, carotid endarterectomy, extensive thyroid surgery with extension of the tumor to the main trunk of the vagal nerve).

When the etiology was due to iatrogenic/surgical intervention with both operative risk to the RLN and main vagus trunk, operative notes were reviewed to clarify the level of vagal injury.

Instrumental swallow parameters were gathered, including (1) type of test (FEES or MBSS/VFSS); (2) timing of testing from onset of paralysis; (3) premature spillage of any texture to vallecula or pyriform sinus; (4) penetration; (5) aspiration; (6) PAS score during thin liquid trial; and (7) residual bolus material (residue) in the vallecula, pyriform sinus, or postcricoid with either puree or solid trials. Diet recommendations (regular vs. modified diet), presence of feeding tube, and behavioral modifications were recorded.

Patients who underwent injection laryngoplasty and had repeat instrumental swallow testing following the procedure were included in a subgroup analysis. Patients were excluded if the vocal fold paralysis had resolved at the time of testing or if the testing occurred past the typical duration of action of the material injected. Posttreatment swallow parameters and diet recommendations were noted.

### 2.3 | Statistical Analysis

Descriptive statistics (means, standard deviations, ranges for continuous variables; counts and percents for categorical variables) were used to summarize the cohort overall and by vagal group. Fisher’s exact tests and *t*-tests (or Wilcoxon rank sum test for length of paralysis) were used to compare the high and low vagal groups for categorical and continuous variables, respectively. For the subset of patients who had injection laryngoplasty, exact McNemar’s tests were used to compare pre- and posttreatment outcomes. *P* < 0.05 were considered statistically significant. SAS V9.4 (SAS Institute Inc., Cary, NC) was used for the analysis.

## 3 | Results

Ninety-six patients met inclusion criteria: 48% (*n* = 46) females and 52% (*n* = 50) males. Twenty-nine percent (*n* = 28) were classified as high vagal UVFP and 71% (*n* = 68) as low vagal UVFP. The high and low vagal cohorts had similar age, sex, race, comorbidities, EAT-10 scores, duration of paralysis, side of paralysis, and presence of glottal gap (Table 1). Iatrogenic injury was the leading cause of UVFP (63%, *n* = 60), followed by tumor compression (13%, *n* = 12). While iatrogenic injury remained the leading diagnosis in the subgroup analysis, CVA (36%, *n* = 10) and intubation injury (13%, *n* = 9) were secondary causes in the high and low vagal groups, respectively. All patients with tongue deviation (5%, *n* = 5) were in the high vagal group. Twenty percent (*n* = 19) of patients had pooling of saliva in the pyriform sinus, more commonly in the high vagal group (57% [*n* = 16] vs. 4% [*n* = 3], *p* < 0.001) (Table 1).

### 3.1 | High vs. Low Vagal Unilateral Vocal Fold Paralysis Dysphagia Characteristics

More patients underwent FEES (68%, *n* = 65) than MBSS/VFSS (32%, *n* = 31), with a similar distribution between high (54% [*n* = 15] FEES) and low vagal (74% [*n* = 50] FEES) groups (*p* = 0.04). Median time from paralysis onset to instrumental testing was comparable across groups: 5.0 months (IQR 1.1–21.8) overall, 5.2 months (IQR 1.4–24.0) in the high vagal group, and 5.0 months (IQR 1.1–15.6) in the low vagal group (*p* = 0.72).

**FIGURE 1 |:**
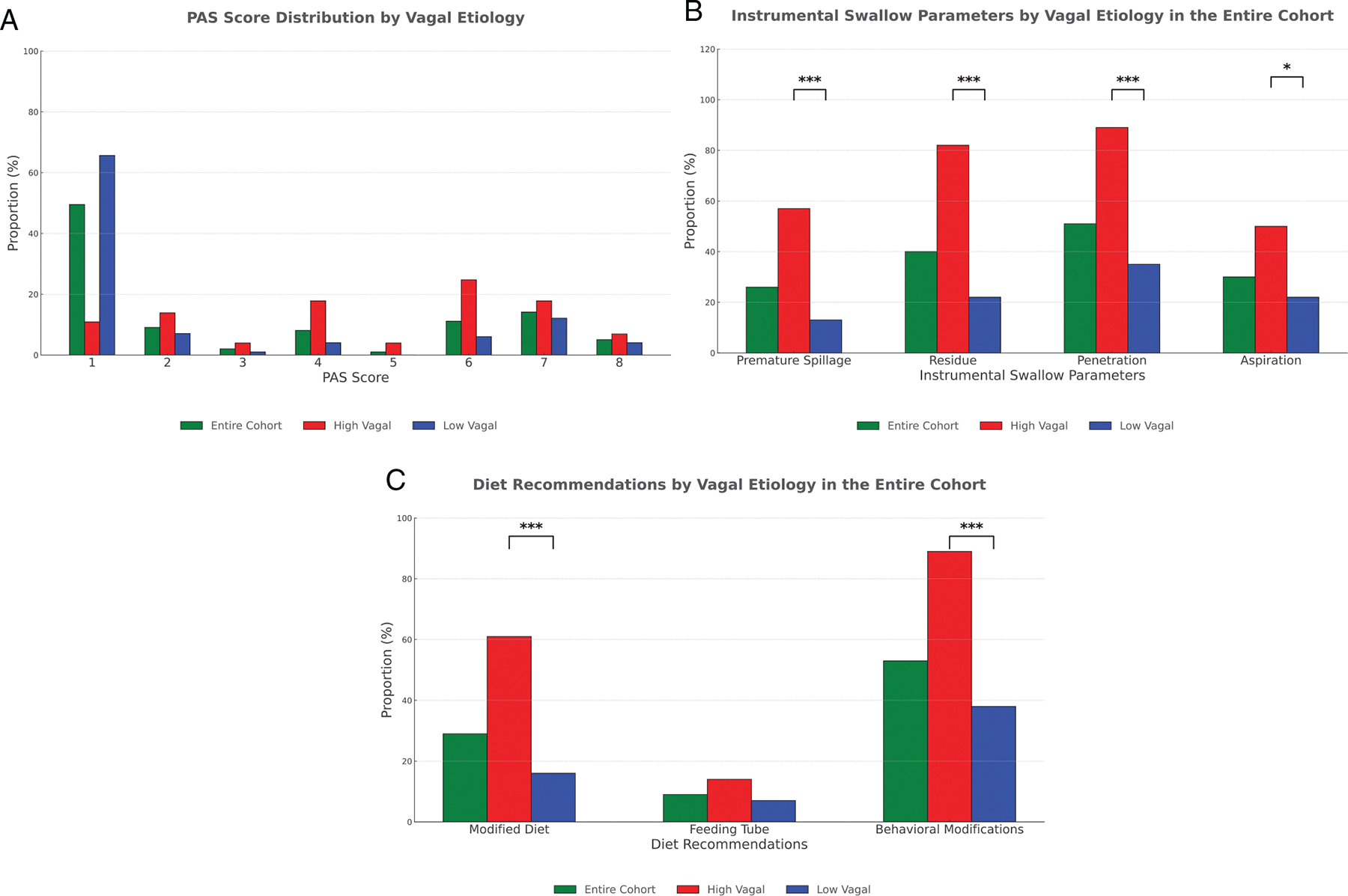
Unilateral vocal fold paralysis swallow characteristics: All patients. (A) Penetration Aspiration Score (PAS) distribution by level of vagal injury in the entire cohort. PAS score of 1 was most common in the overall cohort and in the low vagal group; PAS score of 6 was most common in the high vagal group. (B) All parameters measured by instrumental swallow testing had higher rates of abnormalities in the high vagal group compared to the low vagal group. *p < 0.05; ***p < 0.001. (C) Modified diet and behavioral modifications were significantly more common in the high vagal group compared to the low vagal group. Rates of feeding tube dependence were low overall and similar between groups. ***p < 0.001.

Instrumental swallow parameters differed between the two cohorts. While the PAS score of 1 was most common overall, the two cohorts had different distributions (Figure 1A). Twenty-six percent (*n* = 25) of patients had premature spillage, more frequently in the high vagal group (57% [*n* = 16] vs. 13% [*n* = 9], *p* < 0.0001). Similarly, 40% (*n* = 38) of the overall patients had residue, with a higher prevalence in the high vagal group (82% [*n* = 23] vs. 22% [*n* = 15], *p* < 0.0001). Fifty-one percent (*n* = 49) of patients had penetration and 30% (*n* = 29) of patients had aspiration, both more common in the high vagal group (89% [*n* = 25] vs. 35% [*n* = 24]; *p* < 0.0001) and (50% [*n* = 14] vs. 22% [*n* = 15]; *p* = 0.01), respectively (Figure 1B).

Diet modifications were recommended in 29% (*n* = 28) of patients, more often in the high vagal group (61%, [*n* = 17] vs. 16%, [*n* = 11]; *p* < 0.0001) (Figure 1C). Nine percent (*n* = 9) of patients had a feeding tube, with a similar distribution between the two groups. Behavioral modifications were advised for 53% (*n* = 51), again more frequently in the high vagal group (89% [*n* = 25] vs. 38% [*n* = 26], *p* < 0.0001). These included chin tuck, head turn, effortful swallow, slow pace, small sips, small bites, alternative bites with sips, taking liquids via teaspoon only, avoiding straws, minimizing distractions, and limiting talking while eating.

### 3.2 | Subgroup Analysis: Pre- and Posttreatment Dysphagia Characteristics in High and Low Vagal UVFP Treated With Injection Laryngoplasty

Thirty-two patients underwent injection laryngoplasty with follow-up instrumental swallow evaluation; one was lost to follow-up, leaving 31 patients for analysis (52% [*n* = 16] high vagal, 48% [*n* = 15] low vagal). The groups were similar in age, sex, race, comorbidities, pretreatment EAT-10 score, duration of paralysis, side of paralysis, and presence of glottal gap (Table S1). Pooling of saliva was more frequent in the high vagal cohort (69% [*n* = 11] vs. 13% [*n* = 2], *p* = 0.003). Type and timing of instrumental testing, PAS score, rate of premature spillage, penetration, and aspiration were comparable between groups. However, residue was more common in the high vagal group (100% [*n* = 16] vs. 53% [*n* = 8], *p* = 0.002).

**FIGURE 2 |:**
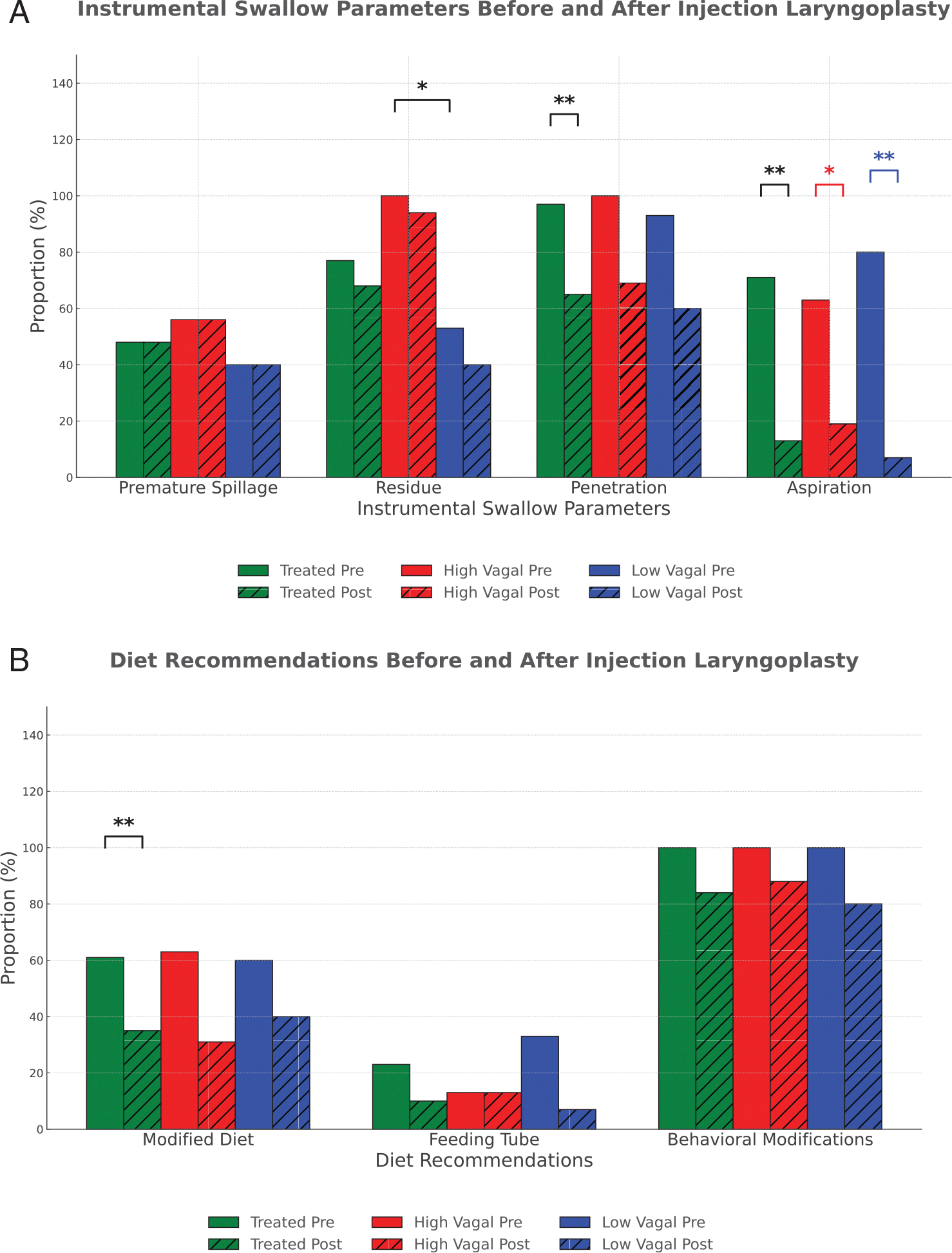
Subgroup analysis: pre- and posttreatment dysphagia characteristics. (A) Pretreatment residue rate was higher in the high vagal group compared to the low vagal group (*p* = 0.002), while rates of premature spillage, penetration, and aspiration were similar. Following treatment, penetration and aspiration rates decreased. *p < 0.05; **p < 0.01. (B) Posttreatment, rates of modified diet recommendations decreased in the overall treated cohort. No statistically significant differences were observed across outcomes within high and low vagal groups. *p < 0.05; **p < 0.01.

Overall, both penetration and aspiration improved posttreatment (97% [*n* = 30] to 65% [*n* = 20], *p* = 0.002) and (71% [*n* = 22] to 13% [*n* = 4], *p* = 0.001), respectively. When categorized by vagal injury level, the prevalence of aspiration (but not penetration) improved in both high and low vagal UVFP (high vagal: 62.5% [*n* = 10] to 19% [*n* = 3], *p* = 0.016; low vagal: 80% [*n* = 12] to 7% [*n* = 1], *p* = 0.001) (Figure 2A). Diet modification rate decreased in the overall treated cohort (61% [*n* = 19] to 35% [*n* = 11], *p* = 0.008), but not in subgroup analysis by vagal injury level. Improvement in behavioral modification did not reach significance in the overall cohort, nor in the subgroup analysis by vagal injury level (Figure 2B).

## 4 | Discussion

### 4.1 | High vs. Low Vagal UVFP Dysphagia Characteristics

Dysphagia in the setting of UVFP ranges in severity, in part due to the level of vagal nerve injury. Dysphagia severity and swallowing profiles differed between patients with high versus low vagal UVFP. In this study, a minority of patients with low vagal UVFP had aspiration (22%) and were recommended a modified diet (16%) and behavioral modifications during meals (38%). Most patients with high vagal UVFP had significantly greater impairment across all parameters, with half presenting with aspiration (50%) and the majority recommended to follow a modified diet (61%) and behavioral modifications (89%).

These findings are concordant with prior smaller studies showing higher residue rates [6], premature spillage [5], and aspiration on MBSS/VFSS [5] in high vagal subgroups. Differences between high and low vagal cohorts highlight greater impairment with coordination between the oral and pharyngeal phases of swallowing—evidenced by premature spillage—and weakened pharyngeal contractility—reflected by bolus residue. Overall, both groups demonstrated penetration and aspiration, consistent with glottal incompetence; however, the prevalence of both penetration and aspiration was higher in the high vagal group, possibly reflecting greater sensory deficits in the pharynx and larynx [20].

Clinical and functional outcomes were more restrictive in the high vagal group, consistent with prior studies showing a high feeding tube dependence rate in high vagal patients (24%–40%) [6, 10] and a high rate of diet modification in a high vagal group compared to a low vagal group (93% vs. 15%) [6]. In the present study, the rate of feeding tube dependence was similar between the two cohorts, possibly because of anatomical changes postesophagectomy, including spit fistula, present in a few patients in the low vagal cohort. Relative to aspiration rate, diet recommendations were more restrictive in the high vagal group compared to the low vagal group. For example, while 22% of patients had aspiration in the low vagal group, only 16% were recommended a modified diet, whereas 50% of patients had aspiration in the high vagal group, but a higher proportion (61%) of patients were recommended to have a modified diet. A possible explanation for this discrepancy is that additional swallowing factors beyond airway protection may have differed between groups. This finding emphasizes that clinical recommendations should be informed by a thorough evaluation of deglutitive physiology at all phases of the swallow and should address how the altered physiology influences swallowing safety and/or efficiency, rather than simply responding to a binary finding, such as the presence or absence of airway invasion.

### 4.2 | Pre- and PostTreatment Dysphagia Characteristics in High and Low Vagal UVFP Treated With Injection Laryngoplasty

The subgroup of patients who underwent injection laryngoplasty demonstrated similar dysphagia characteristics in both high and low vagal UVFP, with a majority having aspiration (63%: high vagal; 80%: low vagal) and being recommended diet modification (63%: high vagal; 60%: low vagal) with universal (100%) behavioral modifications during meals. Swallow function improvements following injection laryngoplasty were similar regardless of etiology. Despite improvements in aspiration and diet modification, most patients (> 80%) were still recommended to follow behavioral modifications even after injection laryngoplasty, regardless of the vagal level of UVFP.

While rates of premature spillage, penetration, and aspiration were similar in patients recommended for injection laryngoplasty in both high and low vagal UVFP, the presence of residue remained more common in the high vagal group, likely secondary to inefficient pharyngeal contractility when the injury is above the bifurcation of the RLN. Poor coordination between the oral and pharyngeal phases of swallow with delayed swallow initiation—typically associated with high vagal injury [13]—was also observed in the treated low vagal group, suggesting additional patient-related factors that affect the ability to coordinate and control the bolus flow. Following injection laryngoplasty, aspiration rates improved as expected due to improved glottal closure, cough efficiency and airway clearance. At the same time, minimal changes were seen in residue and premature spillage, since medialization does not restore coordination and pharyngeal contraction.

Previous studies reported reductions in feeding tube dependence [10] but limited diet liberalization [17] in high vagal subgroups. Coulter et al. performed a systematic review and meta-analysis of medialization treatment for vocal fold paralysis [14]. Though vagal level was not explicitly examined, pooled data suggested diet posttreatment in high vagal groups, though quantitative changes were not detailed [14]. In the present study, aspiration improved in both high and low vagal groups after injection laryngoplasty. Though diet and behavioral modification improvements did not reach statistical significance, potentially due to sample size, the improvements are still clinically relevant. Further, though patients with high vagal UVFP were more likely to present with severe dysphagia, some patients with low vagal etiology had severe dysphagia as well, and both groups benefited from injection laryngoplasty. The persistence of behavioral modifications posttreatment likely reflects that clinical decision making is guided by the patient’s overall clinical presentation rather than solely by the level of vagal injury and associated swallow dysfunction.

This study had several limitations, including its retrospective nature, sample size, and inclusion bias, given that only patients with instrumental swallow testing were included. FEES and MBSS/VFSS findings were used interchangeably during this study; however, they are different types of objective swallow testing with FEES being limited by the inability to assess upper esophageal sphincter and cervical esophageal function. Additionally, the instrumental swallow testing (FEES/MBSS) was completed by multiple individuals within the swallow SLP department, which potentially could lead to minor inter-rater variability, but is representative of real-life clinical practice. High versus low vagal UVFP classification was based on clinical history rather than laryngeal EMG testing. Although the cohorts had a similar distribution of vocal fold paralysis duration and timing of swallow evaluation—typically within 6 months of paralysis onset—clinical outcomes may have been influenced by the natural course of recovery. However, all patients who had treatment did have persistent UVFP on flexible laryngoscopy at the time of posttreatment objective testing. Also, a fraction of patients in the high vagal cohort subgroup did have multiple cranial nerve neuropathies possibly accounting for worse swallowing outcomes. Lastly and importantly, the effect of swallow therapy was not independently assessed and may confound outcome interpretation, as improvements attributed to injection laryngoplasty could also reflect contributions from concurrent swallow therapy in treated patients.

This study contributes a large cohort and great detail on instrumental and clinical swallow characteristics to the literature on dysphagia in UVFP, providing a multidimensional assessment of swallow function with an emphasis on clinical and functional outcomes, and a direct comparison of posttreatment results between high and low vagal UVFP. The findings highlight that instrumental swallow evaluation represents only one component of dysphagia assessment in UVFP and underscore the importance of reporting functional clinical outcomes. Future research should aim to identify predictors of poorer functional swallowing outcomes in UVFP patients. Additionally, systematic investigation of the interactions between the level of vagal injury, treatment strategies, and clinical outcomes is warranted.

## 5 | Conclusion

Patients with high vagal paralysis have a higher prevalence of abnormal instrumental swallow evaluation findings, a more restrictive diet, and a higher need for behavioral modifications, compared with patients with low vagal UVFP. Both high and low UVFP patients who underwent injection laryngoplasty had similar dysphagia profiles. Injection laryngoplasty improved the prevalence of aspiration regardless of vagal injury level, but many patients continued to require behavioral modifications posttreatment. Future studies should aim to identify predictors of poorer functional swallowing outcomes in UVFP patients.

## Supplementary Material

Supplement

Supporting Information

Additional supporting information can be found online in the Supporting Information section. Table S1: Demographics, clinical presentation, and flexible laryngoscopy findings in patients treated with injection laryngoplasty. IQR—interquartile range; SD, standard deviation.

## Figures and Tables

**TABLE 1 |:** Demographics, clinical presentation, and flexible laryngoscopy findings in all patients.

		All patients, *n* (%)	High vagal etiology, *n* (%)	Low vagal etiology, *n* (%)	*p*-Value for comparison of high vs. low vagal groups

Demographics					
*n*		96 (100%)	28 (29%)	68 (71%)	
Age	Mean (SD)	57.9 (16.6)	59.4 (17.5)	57.3 (16.3)	0.58
	Range	19–88	23–88	19–83	
Sex	Female	46 (48%)	14 (50%)	32 (47%)	0.83
	Male	50 (52%)	14 (50%)	36 (53%)	
Race	Asian	6 (6%)	1 (4%)	5 (7%)	0.43
	Black	2 (2%)	1 (4%)	1 (2%)	
	White	83 (87%)	26 (93%)	57 (84%)	
	Prefer not to answer	5 (5%)	0	5 (7%)	
Patient comorbidities					
Charlson Comorbidity Index	Mean (SD)	3.8 (3.1)	4.3 (3.3)	3.6 (3.0)	0.31
Patient-reported dysphagia					
EAT-10	Mean (SD)	11.8 (9) (*n* = 58)	12.7 (10.2) (*n* = 17)	11.4 (8.6) (*n* = 41)	0.68
Physical exam findings					
Oral deficits		5 (5%)	5 (18%)	0	***0.02***
Flexible laryngoscopy findings					
Side of paralysis	Left	61 (64%)	16 (57%)	45 (66%)	0.49
	Right	35 (36%)	12 (43%)	23 (34%)	
Glottal gap		64 (67%)	22 (79%)	42 (62%)	0.15
Pooling of saliva		19 (20%)	16 (57%)	3 (4%)	***< 0.0001***
Vocal fold paralysis characteristics					
Duration of paralysis (months)	Median (IQR)	4.0 (1.8–20)	4.0 (2–24)	4.5 (1.3–15)	0.80
Etiology	Cerebrovascular accident	10 (10%)	10 (36%)	0	***< 0.0001***
	Tumor compression	12 (13%)	5 (18%)	7 (10%)	
	Idiopathic	5 (5%)	0	5 (7%)	
	Intubation	9 (9%)	0	9 (13%)	
	Iatrogenic	60 (63%)	13 (46%)	47 (69%)	
Iatrogenic subgroups	Craniotomy	9 (9%)	9 (32%)	0	
	Esophageal surgery	2 (2%)	0	2 (3%)	
	Neck surgery	29 (30%)	4 (14.3%)	25 (37%)	
	Thoracic surgery	20 (21%)	0	20 (29%)	

*Note:* Neck surgery: High vagal (completion thyroidectomy with high vagal nerve resection, paraganglioma resection with vagus nerve sacrifice, vagal schwannoma with resection); low vagal (Anterior Cervical Discectomy and Fusion (ACDF), thyroid surgery, parathyroidectomy). Bold values indicates statistically significant *p* < 0.0001.

Abbreviations: IQR, interquartile range; SD, standard deviation.

## Data Availability

The data that support the findings of this study are available on request from the corresponding author. The data are not publicly available due to privacy or ethical restrictions.
