# Supplementary material for: Dysphagia Characteristics in High Versus Low Vagal Unilateral Vocal Fold Paralysis
Source: Laryngoscope. 2025 Oct 30;136(4):1800–7. doi: 10.1002/lary.70229 (PMC12629264; doi:10.1002/lary.70229)
Supplement: Supplementary file 1 — Table S1: Demographics, clinical presentation, and flexible laryngoscopy findings in patients treated with injection laryngoplasty. IQR—interquartile range; SD, standard deviation. [file LARY-136-1800-s001.docx]

**Supplement**

**Supplemental Table 1 - Demographics, clinical presentation, and flexible laryngoscopy findings in patients treated with injection laryngoplasty**

|  | | | **All Patients**  **n (%)** | **High Vagal Etiology**  **n (%)** | **Low Vagal Etiology**  **n (%)** | ***P-value for comparison of high versus low vagal groups*** |
| --- | --- | --- | --- | --- | --- | --- |
| Demographics | | | | | | |
| n | |  | 31 | 16 (52%) | 15 (48%) |  |
| Age | | Mean (SD)  Range | 62.2 (17.5)  21-88 | 60.1 (19.8)  23-88 | 64.4 (15.1)  21-78 | 0.50 |
| Sex | | Female  Male | 13 (42%)  18 (58%) | 8 (50%)  8 (50%) | 5 (33%)  10 (67%) | 0.47 |
| Race | | Asian | 2 (7%) | 0 | 2 (13%) | 0.22 |
|  |  | Black | 1(3%) | 1 (6%) | 0 |  |
|  |  | White | 27 (87%) | 15 (94%) | 12 (80%) |  |
|  |  | Prefer not to answer | 1 (3%) | 0 | 1 (7%) |  |
| Patient Comorbidities | | | | | | |
| Charlson Comorbidity Index | | Mean (SD) | 4.4 (3.0) | 4.7 (3.5) | 4.0 (2.5) | 0.54 |
| Patient Reported Dysphagia | | | | | | |
| EAT-10 | | Mean (SD) | 14.4 (8.8)  (n=21) | 13.7 (9.2)  (n=12) | 15.4 (8.7)  (n=9) | 0.66 |
| Physical Exam Findings | | | | | | |
| Oral deficits | |  | 3 (10%) | 3 (19%) | 0 | ***0.017*** |
| Flexible Laryngoscopy Findings | | | | | | |
| Side of paralysis | | Left  Right | 20 (65%)  11 (35%) | 8 (50%)  8 (50%) | 12 (80%)  3 (20%) | 0.14 |
| Glottal gap | |  | 27 (87%) | 15 (94%) | 12 (80%) | 0.33 |
| Pooling of saliva | |  | 13 (42%) | 11 (69%) | 2 (13%) | ***0.003*** |
| Vocal Fold Paralysis Characteristics | | | | | | |
| Duration of paralysis  (months) | | median (IQR) | 2.0 (1-9) | 2.0 (1.5-18) | 2.0 (1-6) | 0.25 |
| Timing of objective swallow testing after treatment | | median (IQR) | 1.3 (0.8-2.5) | 1.3 (1.1-1.9) | 1.0 (0.4-2.52) | 0.47 |
| Etiology | | Cerebrovascular Accident | 5 (16%) | 5 (31%) | 0 | ***0.007*** |
|  |  | Tumor Compression | 3 (10%) | 2 (13%) | 1 (7%) |  |
|  |  | Intubation | 5(16%) | 0 | 5 (33%) |  |
|  | | Iatrogenic | 18 (58%) | 9 (56%) | 9 (60%) |  |
|  | Iatrogenic subgroups | Craniotomy | 8 (26%) | 8 (50%) | 0 |  |
|  |  | Esophageal surgery | 1 (3%) | 0 | 1 (7%) |  |
|  |  | Neck surgery | 5 (16%) | 1 (6%) | 4 (27%) |  |
|  |  | Thoracic surgery | 4 (13%) | 0 | 4 (27%) |  |

*Abbreviations: SD - standard deviation, IQR - interquartile range*
